# A new era in aneuploidy screening: cfDNA testing in >30,000 multifetal gestations: Experience at one clinical laboratory

**DOI:** 10.1371/journal.pone.0220979

**Published:** 2019-08-08

**Authors:** Brittany Dyr, Theresa Boomer, Eyad A. Almasri, Jenna L. Wardrop, Jill Rafalko, Jason Chibuk, Ron M. McCullough

**Affiliations:** 1 Department of Medical Affairs, Sequenom Inc., a wholly owned subsidiary of Laboratory Corporation of America Holdings, Inc., San Diego, California, United States of America; 2 Department of Clinical Science, Sequenom Inc., a wholly owned subsidiary of Laboratory Corporation of America Holdings, Inc., San Diego, California, United States of America; Shanghai Jiao Tong University, CHINA

## Abstract

Since introducing cell-free DNA screening, Sequenom Laboratories has analyzed over 1 million clinical samples. More than 30,000 of these samples were from multifetal gestations (including twins, triplets and higher-order multiples). The clinical laboratory experience with the first 30,000 multifetal samples will be discussed. Maternal plasma samples from multifetal gestations were subjected to DNA extraction and library preparation followed by massively parallel sequencing. Sequencing data were analyzed to identify autosomal trisomies and other subchromosomal events. Fetal fraction requirements were adjusted in proportion to fetal number. Outcome data, when voluntarily received from the ordering provider, were collected from internal case notes. Feedback was received in 50 cases. The positivity rate in multifetal samples for trisomy 21 was 1.50%, 0.47% for trisomy 18, and 0.21% for trisomy 13. Average total sample fetal fraction was 12.2% at a mean gestational age of 13 weeks 6 days. Total non-reportable rate was 5.95%. Estimated performance based on ad hoc clinical feedback demonstrates that possible maximum sensitivity and specificity meet or exceed the original performance from clinical validation studies. Cell-free DNA (cfDNA) screening provides certain advantages over that of conventional screening in multifetal gestations and is available in higher-order multiples.

## Introduction

In the decades leading up to the introduction of cell-free DNA (cfDNA) screening in 2011, there was a marked increase in the rate of multifetal pregnancies. Between 1980 and 2009, the rate of twin births increased 76% [1] and the rate of triplets and high-order multiple births increased more than 400% [2]. The increase in multifetal pregnancies is largely attributed to the expanding use of assisted reproductive technology (ART) [3] and the upward trend of maternal ages due to delayed childbearing [4]. In most multifetal pregnancies, a woman’s age-related risk for aneuploidy is elevated above that of a woman of the same maternal age carrying a singleton pregnancy. [5] Traditionally, maternal age greater than 35 years has been used as an increased aneuploidy risk threshold termed “advanced maternal age”.

Conventional prenatal screening methods for aneuploidy include maternal age, first trimester ultrasound for nuchal translucency (NT) measurement and presence or absence of nasal bone, second trimester ultrasound assessment for aneuploidy markers, and first and second trimester maternal serum screening. Biochemical screening methods have significant limitations, often providing a “pseudo-risk”, and yielding lower sensitivity and specificity in multifetal gestations compared to singletons.[6–8] In twins, first trimester screening combined with NT has a sensitivity for trisomy 21 between 86–87% and a false positive rate (FPR) of 5%, and second trimester screening has an average detection rate of 50–65% with FPR as high as 10%.[9] While maternal age, NT measurement, and ultrasound markers for aneuploidy risk assessment is available in triplets and higher-order multiples, detection of aneuploidy with biochemical screening is not an available option.

The advent of cfDNA testing for use in pregnancy has ushered in a new era of screening. Clinical validation studies in singleton pregnancies have established the high sensitivity and specificity of cfDNA screening [10–16], as well as, increased detection rates, lower FPR, and higher positive predictive values (PPV) over that of conventional screening methods.[17] These factors may have prompted several key organizations, such as the American College of Obstetricians and Gynecologists (ACOG) [18], the Society for Maternal Fetal Medicine (SMFM)[19], the American College of Medical Genetics and Genomics (ACMG)[20], the National Society of Genetic Counselors (NSGC)[21], and the International Society for Prenatal Diagnosis (ISPD)[22] to endorse cfDNA screening as a routine screening option for many singleton pregnancies. In singletons, the sensitivity of cfDNA screening for trisomy 21 is 99%, with a FPR less than 0.02%.[10] While there is still limited data available for performance of cfDNA screening in twins, and relatively no data for higher order multiples, in 10 published twin cohorts [23–32] the sensitivity for trisomy 21 was 92–100% with a FPR less than 0.5%. The improved sensitivity and lower FPR of cfDNA screening for Trisomy 21, compared to conventional screening in multiple gestation pregnancies, positions cfDNA as a valuable screening alternative.

Sequenom Laboratories has analyzed over 1 million MaterniT21 PLUS samples. From 2011–2017, approximately 4% of samples were from multifetal gestations (including twins, triplets and higher-order multiples), which is greater than the rate of multiple births in the US [1], suggesting that providers are turning to cfDNA for aneuploidy screening for multifetal gestations, even in the absence of strong support from professional societies. Here we report on the clinical laboratory experience with the first 30,000 multifetal samples.

## Materials and methods

All data contained within this study were generated from clinical samples received in our Clinical Laboratory Improvement Amendments (CLIA) certified and College of American Pathologists (CAP) accredited laboratory from October 2011 to December 2017. All samples were tested for trisomy 21 as well as presence or absence of chromosome Y. Beginning in February 2012 all samples were also tested for trisomy 18 and trisomy 13. Select samples were opted in for ‘Enhanced Sequencing’ by their ordering healthcare provider for seven common microdeletions associated with eight syndromes: 22q deletion (DiGeorge syndrome), 5p deletion (Cri-du-chat syndrome), 15q deletion (Prader-Willi syndrome/Angelman syndrome), 1p36 deletion syndrome, 11q deletion (Jacobsen syndrome), 8q deletion (Langer-Giedion syndrome), and 4p deletion (Wolf-Hirschhorn syndrome). Trisomy 16 and 22 were also analyzed for additional chromosomal events as part of the Enhanced Sequencing Series.

This study analyzed extracted cfDNA fragments from maternal plasma, which were then subjected to genome-wide sequencing and algorithmic analysis for chromosomal aneuploidies and subchromosomal under-representation in specified regions, when requested. Both fetal (placental) and maternal fragments were sequenced and mapped to unique regions of the genome. The unique reads were assigned to a 50 kb bin, normalized across the genome, and counted. An under- or over-representation of fragments in a 50 kb bin are indicative of a loss or gain in the genome profile respectively. For autosomal trisomy analysis, this technique analyzes for over-representation of DNA along the entire chromosome as previously described. [14] To determine the presence of subchromosomal deletions, we used a statistical method to search for consistently under-represented regions followed by a decision tree to differentiate whole-chromosome events from deletions. [33]

Fetal fraction requirements for multiples were adjusted in proportion to fetal number (e.g. twice singleton minimum threshold for twins, three times minimum singleton threshold for triplets, etc.). It should be noted that the methodology used to estimate fetal contribution and minimum threshold values have evolved over time, spanning several assay enhancements. [34–35] However, regardless of the time stamp or method employed, the proportional stringency increase for amplified fetal fraction to fetal signal has remained constant.

Data from all samples was reviewed by a laboratory director prior to the final reporting of results to the ordering provider. Samples with insufficient fetal DNA were classified as quantity not sufficient (QNS) using a previously described method. [34–35] Samples failing other laboratory quality metrics including library and sequencing passing criteria were classified as technical non-reportable. Statistical analysis of the multifetal screening cohort employed a two-sample, two-sided proportional z-test to compare non-reportable rates and average risk samples, which are samples submitted with no high risk indication such as advanced maternal age or abnormal ultrasound finding, from 2011–2016 to 2017: z scores = 10.4 and -37.3 respectfully; p values <0.001. A two-sample, two-sided modified t-test was used to compare turnaround time from 2011–2016 to 2017: t score = 28.0, df = 30,378, p-value <0.001, F = 1.95.

Accuracy of test results reported by CLIA certified and CAP accredited laboratories is an important metric to track, however complete individual outcome data is often difficult to obtain, especially in a high throughput clinical laboratory setting. To assess maximum possible sensitivity and specificity, we compiled all of the *ad hoc* feedback we received regarding confirmed results; true positives, true negatives, false negatives, and false positives from ordering providers through June 2018. These outcome data were provided voluntarily by ordering clinicians, and typically based on results of prenatal diagnostic testing such as karyotype and/or microarray or birth outcomes. Based on the feedback we received, the relative sensitivities and specificities were calculated using standard formulas: [sensitivity = true positives / (true positives + false negatives), specificity = true negatives / (true negatives + false positives)] under the assumption that if the lab was not contacted by the clinician, then the results were not discordant.

The anonymized data analyzed for this retrospective study was obtained from existing patient data. All patients signed informed consent prior to testing. All patient data that was generated as a result of the MaterniT21 PLUS LDT test was de-identified and combined for analysis in compliance with FDA Guidance Document “Informed Consent for *In Vitro* Diagnostic Device Studies Using Leftover Human Specimens that are Not Individually Identifiable” issued on April 25, 2006 and the IRB approved protocol SCMM-RND-401 “Compliant Analysis of Patient Samples and Data” and is exempt from IRB review. We are reporting on the overall clinical experience with the assay (positivity rates, non-reportable rates, cohort demographics, etc.), and do not provide identifiable information of the individual cases.

## Results

### The laboratory experience

Between October 2011 and December 2017, 30,826 multifetal samples were submitted to the laboratory for testing. Samples were submitted spanning the entire course of pregnancy with 65% of samples submitted in the first trimester, 33% submitted in the second trimester, and only 2% of samples were submitted in the third trimester. Test metrics and total cohort demographics are shown in Table 1. In 2017 alone, 4,740 multifetal samples were submitted to the laboratory for testing. Samples submitted in 2017 compared to the total cohort had similar mean maternal age, mean gestational age, mean maternal BMI and average fetal fraction. However, in 2017 compared to all multifetal samples from 2011–2016, there was a statistically significant decrease in average turnaround time (from 6.0 calendar days to 4.9 calendar days, p < 0.001), a statistically significant decrease in total non-reportable rate (from 6.55% to 2.66%, p < 0.001), as well as a statistically significant increase in the number of average risk samples (from 3.1% to 16.1% p < 0.001).

**Table 1:** Laboratory performance metrics and patient demographics.

	Total multifetal cohort 2011–2017 (n = 30,826)	Multifetal samples collected in 2017 (n = 4,740)
**Average turnaround time**	5.9 calendar days	4.9 calendar days
**Positive or negative result**	94.05% (28,992)	97.34% (4,614)
**Total non-reportable (QNS + Technical)**	5.95% (1,834)	2.66% (126)
**QNS (low fetal fraction)**	5.29% (1,632)	2.41% (114)
**Technical non-reportable**	0.66% (202)	0.25% (12)
**Mean maternal age**	35.1 years (range 14.5–61.5 years)	34.1 years (range 15.7–61.5 years)
**Mean gestational age**^a^	13 weeks 6 days (range 9–38 weeks)	13 weeks 4 days (range 9–35 weeks)
**Mean maternal BMI**^b^	27.54 kg/m^2^	27.78 kg/m^2^
**Average fetal fraction**	12.2%	12.1%

^a^Gestational age was determined by LMP or ultrasound as provided on the test requisition form (TRF).

^b^Maternal height and weight are not required for testing and thus were not provided for all samples.

Indication for testing was recorded based on information provided on the test requisition form (TRF) by the ordering clinicianNearly two thirds, 65.2%, of tests were ordered due to “advanced maternal age” only. Ultrasound findings, multiple indications, and average risk screening were the next most frequently cited indications consisting of 14.4%, 6.8% and 5.1% of referrals respectively. The least frequently referred indications were personal or family history of aneuploidy and abnormal serum biochemical screening consisting of 3.5% of the referred indications each and 1.6% of referrals citing other high risk indication.

From 2011 to 2013, 5,381 samples were submitted for testing under the designation ‘multifetal’, denoting any fetal number greater than one. Beginning in 2014, the TRF requested a more specific multifetal designation, and 94.3% (23,986) of samples were from twins, 2.8% (709) from triplets, and 2.9% (750) from “other” multifetal gestations. The “other” category has historically been used by clinicians to indicate higher order multiples (quadruplets, etc.), co-twin/triplet/quad demise, and/or selective reduction. Additional breakdown of twin and triplet laboratory metrics are shown in Table 2.

**Table 2:** Sample characteristics and demographic breakdown for twins and triplets.

	Twins (n = 23,986)	Triplets (n = 709)
**Positive or negative result**	93.95% (22,536)	78.70% (558)
**Total non-reportable (QNS + Technical)**	6.05% (1,450)	21.30% (151)
**QNS (low fetal fraction)**	5.47% (1,313)	20.60% (146)
**Technical non-reportable**	0.57% (137)	0.71% (5)
**Mean maternal age**	35.0 years (range 14.5 years– 61.5 years)	35.0 years (range 18.3 years– 49 years)
**Mean gestational age**^a^	13 weeks 5 days (range 9 weeks– 38 weeks)	13 weeks 1 day (range 9 weeks– 33 weeks)
**Mean maternal BMI**^b^	27.64 kg/m^2^	27.59 kg/m^2^
**Average fetal fraction**	12.33%	13.20%

^a^Gestational age was determined by LMP or ultrasound as provided on the TRF.

^b^Maternal height and weight are not required for testing and thus not provided for all samples

### Common aneuploidy results and outcomes

**Fig 1:**
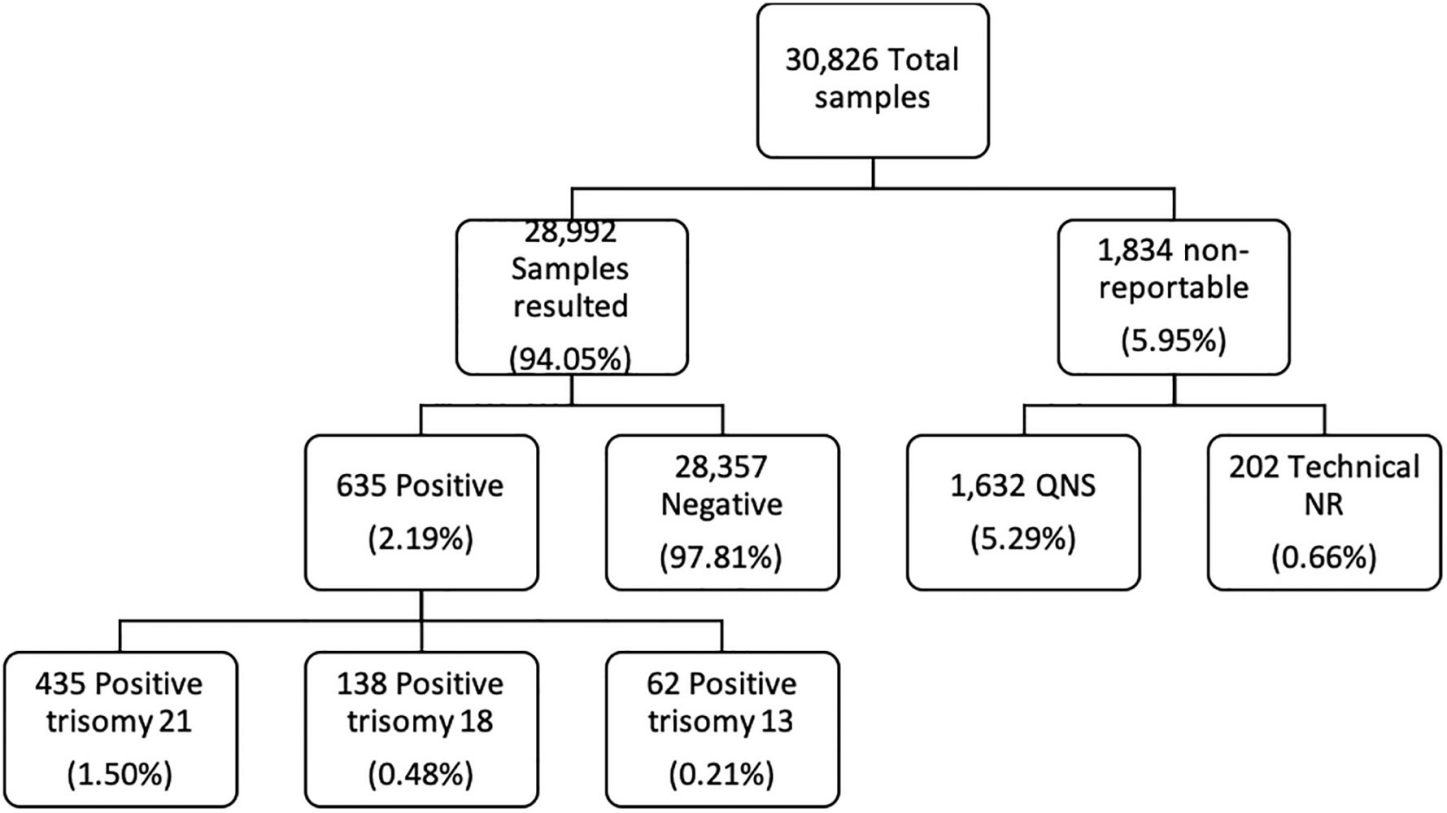
Breakdown of sample analysis.

For cfDNA screening of all multifetal pregnancies, twins, triplets, and higher order multiples, more than 94% of all samples resulted in a simple positive or negative result; additional details regarding these results are provided in Fig 1. The collective positivity rate of resulted samples is 2.19%. Positivity rates for trisomy 21, trisomy 18 and trisomy 13 were 1.5%, 0.48%, and 0.21% respectively.

Further analysis (Table 3) of the positivity rates by indication show that samples with multiple indications and abnormal ultrasound findings, collectively >21% of the total sample population represented a higher percentage of the positive sample cohort per indication, collectively > 40%. In the total sample cohort the referral for advanced maternal age accounts for >65% of all samples while in the positive sample cohort it accounts for just over 55% of samples. Samples submitted as average risk screening, no high risk indication reported, comprised 5.1% of the total sample cohort and only 0.8% of all positives reported.

**Table 3:** Positivity rate and percent of total positives by clinical indication.

Indication for testing	Positivity rate	Percent of total positives (n = 635)
**Multiple Indications**	5.42% (113/2,084)	17.80%
**Ultrasound Finding**	3.20% (142/4,439)	22.36%
**Maternal Age**	1.74% (350/20,115)	55.12%
**Serum Biochemical Screening**	1.40% (15/1,069)	2.36%
**Personal/Family History**	0.65% (7/1,082)	1.10%
**Other High Risk**	0.63% (3/479)	0.47%
**Average Risk**	0.32% (5/1,562)	0.79%

As expected, the majority of the outcome data received were notifications of false positive and false negative results. Confirmed results are less frequently voluntarily communicated to the laboratory. In total, we received communication from providers with 50 outcomes. The limited clinical outcome feedback we received from clinicians through June 2018 allows an estimated maximum possible sensitivity and specificity calculation and is provided in Table 4.

**Table 4:** Multifetal performance of core trisomies based on ad hoc feedback.

Chromosome	21	18^a^	13^a^
**Reported as negative**	28,561	28,814	28,887
**Reported as positive**	435	138	62
**Communicated true positives**	16	8	3
**Communicated false positives**	4	1	7
**Communicated false negatives**	7	4	0
**Relative observed sensitivity**^b^	98.40%	97.16%	>99.99%
**Relative observed specificity**^c^	99.99%	>99.99%	99.98%
**Relative observed positive predictive value**^d^	99.08%	99.28%	88.71%

^a^Screening for trisomy 13 and trisomy 18 began in February 2012

For the purposes of these calculations all positives were considered true unless reported as false positive and all negatives were considered true unless reported as false negative from the ordering clinician.

^b^observed sensitivity = true positives / (true positives + false negatives)

^c^observed specificity = true negatives / (true negatives + false positives)

^d^observed positive predictive value = true positives / all positives

### Microdeletion and additional autosomal aneuploidy results and outcomes

Of all samples that received a result, over 58% were opted in to ‘Enhanced Sequencing’ by the ordering healthcare provider for additional screening of eight microdeletion syndromes as well as screening for trisomy 16 and trisomy 22. Of the 16,951 samples that opted into additional screening, a total of six positive results for microdeletions and seven positive results for trisomy 16 were reported in this multifetal cohort. In total, four of the six positively reported microdeletions pursued diagnostic testing reporting one false positive and three true positives reported back to the laboratory (Table 5). The two remaining positively reported microdeletions did not undergo diagnostic testing; however, the results were considered likely concordant due to clinical findings consistent with the suspected microdeletion syndrome.

**Table 5:** Clinical details of positive microdeletions on cfDNA screening in multifetal gestations.

Positive microdeletion on cfDNA screen	Indication for screening	Gestational age (weeks)	Diagnostic testing	Accuracy of cfDNA results
**del22q11**	Maternal Age and Ultrasound Finding	28	Confirmed postnatal diagnosis	True positive
**del22q11**	Ultrasound Finding	34	Declined diagnostic testing	Suspected positive due to clinical findings
**del5p15**	Maternal Age	10	Confirmed by prenatal diagnosis	True positive
**del8q24**	Maternal Age	11	Negative prenatal diagnosis	False positive
**del8q24**	Maternal Age	14	Declined diagnostic testing	Suspected positive due to clinical findings
**del15q11**	Other high risk	13	Confirmed prenatal diagnosis	True positive

Of the seven cases reported positive for trisomy 16, six cases, 86% reported a spontaneous abortion or co-twin demise after screening. Two cases reported a false positive result back to the laboratory based on normal karyotype analysis from amniocentesis. In one false positive trisomy 16 case there was known co-twin demise after screening and the amniocentesis was performed on the surviving fetus. In the other false positive trisomy 16 case intrauterine growth restriction was reported, a common finding in cases of confined placental mosaicism (CPM). In both false positives for trisomy 16, chorionic villus sampling (CVS) and placental biopsy after delivery were not performed and therefore no additional information was received about the placental genetic makeup.

## Discussion

This retrospective study is the largest report to date, of cfDNA screening experience in twin pregnancies, and the only known experience in triplets and higher-order multiples. While the absence of outcome data is a limitation, this series of over 30,000 multifetal pregnancies analyzed with cfDNA screening shows that the assay performs reliably and compares favorably to the laboratory experience with singleton gestations. This data set provides a general sense of cfDNA screening performance in multifetal pregnancies for the clinical community. In this multifetal cohort compared to our initial clinical experience with the first 100,000 MaterniT21 PLUS LDT samples [36] (comprised of singleton and multifetal samples); average maternal age was the same, 35.1 years, but there was a shift in timing of screening with significantly more samples being submitted in the first trimester, 65% vs 54%. Unsurprisingly, this was also represented by a lower mean gestational age in the ‘multifetal only’ cohort (13 weeks 6 days vs. 15 weeks 3 days in the first 100,000 samples). This suggests that providers and patients may be opting for earlier screening to allow more time for prenatal diagnosis, and ultimately, information gathering for pregnancy management or decision making in the event of a positive cfDNA screen.

Laboratory process improvements are reflected by significant reductions in turnaround time, from 6.97 days in the first 100,000 samples [36], to 4.9 days in 2017, a 29.7% decrease. There was also a 34% decrease in the rate of technical failures from 1% to 0.66%. Assay improvements, such as sample specific fetal fraction, have led to statistically significant improvements in non-reportable rates. From 2011–2016 the total non-reportable rate for multifetal samples was 6.55% compared to 2.66% in 2017. There is a higher QNS non-reportable rate or low fetal fraction rate in the multifetal cohort compared to the mostly singleton cohort, 5.29% vs. 0.9%. This marked increase in QNS non-reportable results can be explained by more stringent fetal fraction requirements in place for multifetal gestations. The effect of higher fetal fraction thresholds based on the fetal count to the QNS rate is also clearly demonstrated within the current population by higher QNS non-reportable results in triplets vs. twins (20.60% vs 5.47%) even though average fetal fraction is 7% higher in the triplet cohort over the average twin fetal fraction (Table 2). Additionally, as also reported in the McCullough et al. [36] cohort of 100,000 samples, a review of the current study population of low fetal fraction samples showed a higher proportion of samples with elevated BMI compared to those samples that received a result. In the current study population there was a 20% increase in average BMI in the QNS non-reportable group compared to samples with a successful initial result, 34.17 kg/m^2^ to 27.13 kg/m^2^ respectively.

Compared to the first 100,000 MaterniT21 PLUS samples [36], where 11.3% of samples were submitted subsequent to abnormal maternal serum screening, in the multifetal study population only 3.5% of samples were submitted with an indication of abnormal maternal serum screening. This metric could be indicative of more providers choosing cfDNA over conventional screening in the multifetal population. Furthermore, maternal serum screening is not available for pregnancies involving higher-order multiples.

The small number of samples positive for a microdeletion in this cohort is not surprising given the sample size (n = 16,951) respective to low population incidence for many of the syndromes. For example, most of the microdeletion syndromes for which no cases were identified the expected incidence is approximately 1 in 20,000 to 1 in 50,000. [37] This limited data set precludes determination of detection rates of microdeletions in multifetal pregnancies. Ultimately, though, five of the six reported microdeletions were confirmed or highly suspected given the clinical picture. In addition, no false negative results for microdeletions were reported back to the laboratory.

There are additional considerations for cfDNA screening in multifetal pregnancies that may benefit from a genome-wide sequencing platform over more targeted sequencing. One unique factor that may have clinical utility is the ability to perform calculations for a sample-specific mosaicism ratio due to a genome-wide sequencing approach. The concept of a mosaicism ratio (MR) was first introduced by Sequenom Laboratories in 2017 in an effort to better understand the biological origin of discordant cfDNA results, particularly in the presence of discordant clinical feedback, and to better refine the positive predictive value (PPV) of the test. In essence, a mosaicism ratio is generated by dividing the fetal fraction estimated for only the aneuploid chromosome (or microdeletion event) by the fetal fraction estimated for all chromosomes in the sample.

Another clinically useful application of mosaicism ratio in multifetal pregnancies is illustrated by a microdeletion case of a confirmed 15q deletion in a multifetal pregnancy. In follow up discussion with the ordering provider, it was noted by the laboratory that the strength of the signal of the deleted region of chromosome 15 was about half that of the overall fetal fraction, in other words the MR was ~50%. One possible interpretation for this data, especially for singleton pregnancies, is a mosaic event. However, in a multifetal gestation, the more likely answer for this result is only one affected fetus. In this particular case, prenatal diagnosis through amniocentesis sampling of each fetus confirmed only one fetus to be affected.

## Conclusion

In over three quarters of a million samples submitted to one clinical laboratory, approximately 4% of samples were from multifetal gestations, which is greater than the 1 in 30 rate of multiple births in the US. [1] CfDNA screening overcomes some disadvantages of conventional screening in multifetal gestations, including providing a result for trisomies 18 or 13 and the availability of screening in higher-order multiples. CfDNA offers patients with multifetal gestations accurate and reliable screening for fetal aneuploidy that has met or exceeded performance from the original clinical validation studies. It is imperative that laboratories factor in the multifetal status in their quality criteria to ensure similar performance to singleton pregnancies.
